# Distribution Pattern and Influencing Factors of Heavy Metal Resistance Genes in the Yellow River Sediments of Henan Section

**DOI:** 10.3390/ijerph191710724

**Published:** 2022-08-28

**Authors:** Kai Zhang, Kuangjia Li, Minghui Tong, Yangchun Xia, Yongxin Cui, Ziyi Liu, Qi Chen, Qidi Li, Feiyue Hu, Fengxia Yang

**Affiliations:** 1 School of Geographic Sciences, Xinyang Normal University, Xinyang 464000, China; 2 Henan Key Laboratory for Synergistic Prevention of Water and Soil Environmental Pollution, Xinyang Normal University, Xinyang 464000, China; 3 Development Research Center, Ministry of Water Resources of People’s Republic of China, Beijing 100032, China; 4 Agro-Environmental Protection Institute, Ministry of Agriculture and Rural Affairs, Tianjin 300191, China

**Keywords:** heavy metal resistance genes, Yellow River, bioavailable, sediment, potential hosts

## Abstract

The transformation of heavy metal resistance genes (MRGs) in the environment has attracted increasing attention in recent years. However, few studies have reported the MRG content in the Yellow River, one of the main irrigation water sources in the North China Plain. In this study, we quantified MRG abundance by a metagenomic approach, and assessed the influence on MRGs of both bioavailable and total heavy metal (HM) content. The results indicate that Cu-resistant genes are the most common genes, and the prevalence of *ars*M needs more attention. *Comamonadaceae* is the dominant family in the Yellow River, and the presence of organic pollutants may contribute to the prevalence of *Vicinamibacteraceae*, *Nocardioidaceae*, and *Flavobacteriacea*. The results of the Mantel test and Spearman analysis indicate that both the bioavailable fractions and total content of HMs could have little influence on MRGs. Network analysis results indicate that some dominant bacteria could be the potential hosts of some prevalent MRGs, which may exert an adverse impact on human health.

## 1. Introduction

The Yellow River is the second-largest river in China, originating in Qinghai Province and flowing into the Bohai Sea in Shandong Province. The river is the most important one in northern China and plays an important role in daily life for more than 155 million people [1]. The irrigation area of the Yellow River Basin is 5.06 million hectares, accounting for 9.7% of China’s irrigation area. In recent years, various pollutants have been revealed in the river, posing a significant threat to its ecological services [2,3]. Among them, heavy metals (HMs) are one of the most dangerous contaminants due to their consistent toxicity, quality, and proclivity to persist in organisms and transmit food supply chain amplification, and they are also non-degradable [4]. In addition, they can transfer to the surrounding environment through different pathways [5,6]. Acute heavy metal intoxications can have various adverse effects on human health [7].

The content and species of HMs affect the composition and diversity of sedimentary microbial communities in rivers [8]. The ways HMs harm bacteria are diverse, such as disrupting cell wall integrity and interfering with DNA and RNA synthesis, leading to a change in dominant bacteria. For example, He et al. found that *Fontibacter*, *Mariniphaga*, *Symbiobacterium*, and *Thauera* were preferentially enriched in sediments with V, Cr, and Mn pollution [9], and Arroyo-Herra et al. reported that *Proteobacteria*, *Firmicutes*, and *Actinobacteria* were the dominant bacteria in HMs polluted sediments [10]. However, current knowledge about the impacts of HMs on bacterial communities in the Yellow River is limited.

Because surface water is used for irrigation, cultivation, swimming, and fishing, the incidence of resistome in river systems has become a key concern for human health. For example, Ture et al. found that there is a strong positive correlation between MRG levels in fish and the prevalence of MRG-carrying *E. coli* and *coliform*, which would be a problem in aquaculture, aquatic ecosystems, and public health [11]. Thus, the distribution pattern of MRGs and its driven factors have received increasing attention in recent years. For instance, Jie et al. found that MRGs existed widely in river sediments that were severely polluted with heavy metals [12]. Salam et al. investigated the occurrence of MRGs in HM-contaminated soil by using metagenomics, and found a variety of MRGs that expressed resistance to HMs [13].

The relationship between HMs and MRGs has been studied and discussed in previous research. The results concern the role HMs play in the propagation of MRGs [14,15,16]. However, the influence of bioavailable fractions of HMs on MRGs, rather than the total HM content, should be addressed. For this study, we selected the Henan section of the Yellow River as the object, and quantified the heavy metal resistome, bacterial community, and HM fractions by metagenomic analysis, 16S rRNA amplicon sequencing, and inductively coupled plasma optical emission spectrometry. The objectives of this study were to (1) determine the distribution pattern of MRGs, (2) clarify the role of HMs in the propagation of MRGs, and (3) identify the potential hosts of MRGs in the Henan section of the Yellow River. 

## 2. Materials and Methods

### 2.1. Sampling

**Figure 1:**
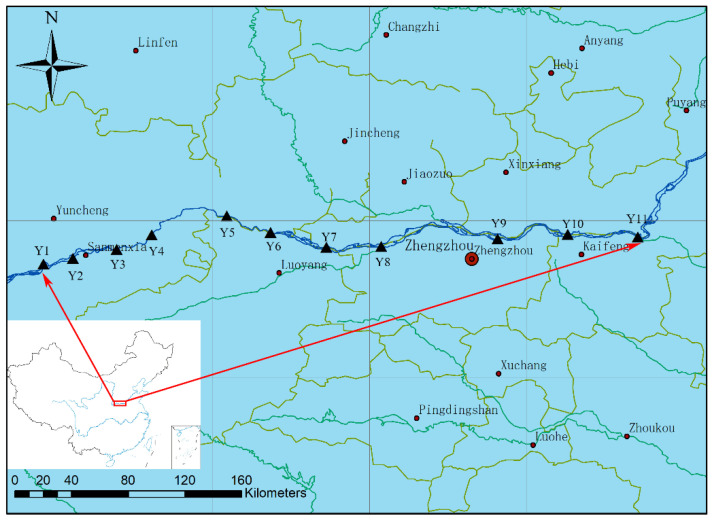
Sample sites in the Henan section of the Yellow River.

Sediment samples were harvested from 11 sites along the main Yellow River corridor in the Henan section, and the sampling was carried out in June 2021. The geographical location of the selected sampling sites is displayed in Figure 1. Y8 was set to assess the influence of tributaries on the main stream of the Yellow River. The surface sediment was collected by using a sediment sampler (KH0201, Xinbao, Jintan, China). Three samples were collected from the ambient locations of each sample site, and these three samples were combined to prepare one sample. All samples were contained in a zip-locked bag and immediately sent to a laboratory in an icebox. Samples were stored at −20 °C until DNA was extracted. 

### 2.2. Speciation Analysis and Content Determination of HMs

Speciation analysis was performed with the BCR approach [17], and the content of 6 HMs (Zn, Pb, Cd, Ni, Mn, Cu) was determined in our previous study [18]. Briefly, acetic acid, hydroxylammonium chloride, hydrogen peroxide, and ammonium acetate were adopted to extract the fraction of B1 (exchangeable fraction), B2 (reducible fraction), and B3 (oxidizable fraction), respectively, and nitrohydrochloric acid was adopted to extract the B4 fraction (residue fraction). An ICP-OES instrument (Agilent 8900, Santa Clara, CA, USA) was adopted to determine fraction content. Data quality control measures included parallel samples, standard reference samples (GSD-5a), and blank samples.

### 2.3. DNA Extraction and Quality Control

Total genomic DNA extraction was performed with a FastDNA Spin Kit for Soil (MP Bio, Santa Ana, CA, USA) according to the manufacturer’s protocol. The integrity and purity of DNA were verified with agarose gel electrophoresis (Tanon 1600, Shanghai, China) and an ultramicrospectrophotometer (Nanodrop 2000, Thermo Fisher, USA), respectively. The DNA content was determined by using a Quantus Fluorometer (Qubit 4.0, Thermo Fisher, Waltham, MA, USA) with the Qubit dsDNA HS Assay Kit (Thermo Fisher, Waltham, MA, USA). Genomic DNA was stored in a refrigerator at −80 °C for further analysis.

### 2.4. 16S Amplicon Sequencing

The hypervariable V3–V4 region of 16S rRNA was amplified by PCR. Detailed information on 16S amplicon sequencing is shown in Supplementary File Text S1. The representative reads were grouped into operational taxonomy units (OTUs) by a similarity of 97% cut-off. OTUs were assigned using the RDP classifier (http://rdp.cme.msu.edu/, accessed on 28 August 2012, version 2.2). The Y11 bacterial community was not determined due to the low DNA content of this sample site.

### 2.5. Metagenomic Analysis

For the construction of a PE library, DNA was fragmented to an average size of approximately 400 bp by adopting the Covaris M220 ultrasonicator (Gene Company Limited, Hong Kong, China). The paired-end library was constructed by NEXTflexTM Rapid DNA-Seq (Bioo Scientific, Austin, TX, USA). Sequencing was carried out by adopting Illumina NovaSeq/Hiseq Xten (Illumina, San Diego, CA, USA) at Majorbio Bio-Pharm Technology Co., Ltd. (Shanghai, China). Low-quality reads (length < 50 bp, quality value < 20, or presence of N reads) were discarded by adopting fastp (https://github.com/OpenGene/fastp, version 0.20.0, accessed on 28 August 2018). Sequence assembling was conducted by using a succinct DE Bruijn graphs principle of stitching software MEGAHIT (https://github.com/voutcn/megahit, version 1.1.2, accessed on 28 August 2016), and the contigs ≥ 300 bp were chosen as the final assembling result. The MRGs were characterized against the BacMet database (http://bacmet.biomedicine.gu.se, accessed on 28 August 2014) using BLASTx (e-value ≤ 1 × 10^−5^), and the parameters were set as matching degree ≥ 85% and ammonia length ≥ 30. The metagenomic analysis was not conducted in H11 due to the low DNA content of this sample site.

### 2.6. Statistical Analysis

All experimental data were statistically analyzed using Microsoft Excel 2019. Spearman correlation analysis was performed using SPSS V22.0 (IBM, Armonk, NY, USA), and *p* < 0.05 was considered statistically significant.

## 3. Results and Discussion

### 3.1. Pollution Level of HMs in the Henan Section of the Yellow River

**Figure 2:**
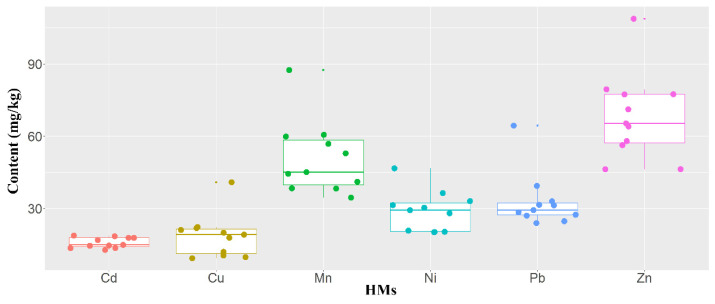
HM content in the Henan section of the Yellow River. The Cd content was multiplied by 100 and the Mn content was divided by 10 to facilitate the visualization of the HM content.

The HM content in the Henan section of the Yellow River was low in general (Figure 2). The toxicity of Cd is much higher than that of the other selected HMs. Thus, this element contributes a large proportion of the potential ecological risk in the Yellow River, although the content of this element is only slightly higher than the background value. Furthermore, the HM content of the Yellow River is lower than that of the other rivers in most cases [18]. These results suggest that the pollution level of HMs is low in the Henan section of the Yellow River. Heavy metals causing soil pollution based on human actions such as mining, steelworks, and electroplating negatively affect human health and ecosystem stabilization [19,20]. In recent years, Henan Province has gradually strengthened the control of soil pollution sources and regulated the sources of HM pollution, such as steel, non-ferrous metals, and dyeing industries [21]. Such policies can help to reduce HM pollution in the Yellow River.

For fraction analysis, the proportion of B1 fraction is lower than that of other environmental media [18]. The highest B1 fraction proportion is Ni, which only accounts for 2.82% of the total content, indicating that the content of the B1 fraction is generally low in the Yellow River. On the contrary, B4 fractions of HMs, which mainly contain minerals and are hard to release into the water to pose a human health risk, are the main contributors to the total HM content.

### 3.2. Distribution Pattern of MRGs in the Henan Section of the Yellow River

We determined 89 MRGs in total, and the detection rate is 45.5%. Eighteen MRGs can be detected in all samples, accounting for 20.2% of the total detected gene number. Additionally, 47 MRGs can be found in ≥50% of samples, accounting for 52.8% of the total detected gene number. These results indicate that MRGs are ubiquitous in the Henan section of the Yellow River. MRGs express resistance to all 11 HMs, and the top three MRGs for a single gene are Cu (17), As (11), and Hg (10). Moreover, there are 32 MRGs that can express resistance to multiple heavy metals. 

**Figure 3:**
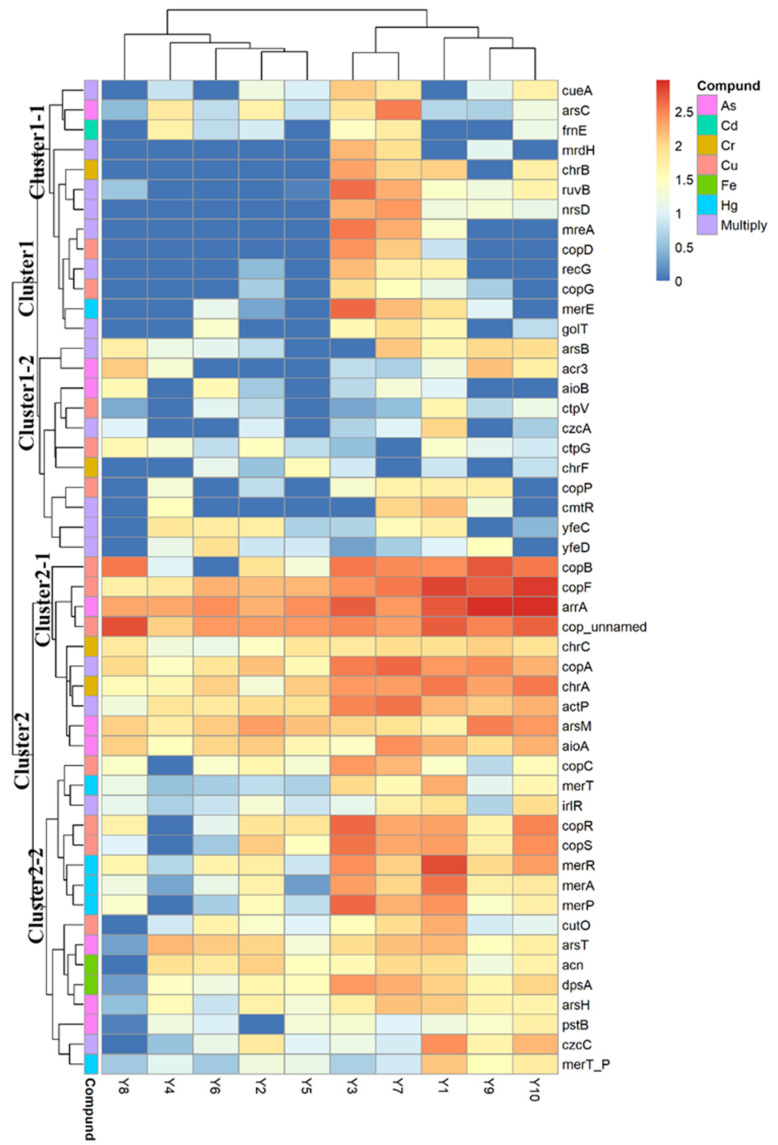
The content of top 50 MRGs in sediments in the Henan section of the Yellow River. The values demonstrated in the figure were calculated as follows: log10(MRG abundance × 10^4^).

The abundance of MRGs ranges from 146.61 (Y4) to 935.84 (Y3) RPKM, and the variation coefficient is 66.07%. Moreover, the clustering results of Figure 3 show that Y3 and Y7 are clustered together and Y1, Y9, and Y10 are clustered together, although their geographical distance is far. These results indicate that MRG content varied greatly among different sample sites of the Yellow River in the Henan section. Considering the modest level of HM pollution, the bacterial community may be the underlying reason causing the obvious MRG abundance difference. Notably, Y8 is where the Sishui River flows into the Yellow River, and the MRG content of this sample point is just the sixth highest. The result indicates that the inflow of tributaries contributes little to the MRG content in the Yellow River.

Cluster analysis was conducted for the top 50 MRGs in the Yellow River (Figure 3). MRGs could be divided into two clusters in general, and both the abundance and detection frequency of cluster 2 were higher than those of cluster 1. Cluster 2 can be further divided into clusters 2-1 and 2-2, which means there are some differences in distribution patterns between these two subclusters. There are 8 of the top 10 MRGs in average abundance from cluster 2-1, and the two lowest abundance genes, *aio*A and *chr*C (detection frequency is 100%), are the 12th and 19th top genes, respectively. These results indicate that the MRGs belonging to cluster 2-1 are the most common genes in the Yellow River of China. Six (*cop*_unnamed, *cop*F, *cop*B, *cop*A, *act*P, and *cop*R) of the top ten MRGs can be resistant to Cu, indicating that Cu-resistant genes are the most common genes. This result is consistent with previous studies, which means the Cu-resistant gene may be the most common in the environment [16,22]. Two As-resistant genes, *arr*A and *ars*M, are also in the top ten genes. The abundance of *arr*A is the highest among all the detected MRGs. The *arr*A gene encodes As (V) respiratory reductase, which is responsible for As (V) reduction; thus, the gene is considered as a reliable marker for As (V) respiration [23,24]. The prevalence of the gene means that there is a strong reduction in As (V) in the Yellow River. The abundance of *ars*M is the fifth highest among all the detected MRGs. *Ars*M is the core enzyme for methylation of As [25], and previous studies indicated that *ars*M can convert As (III) to a more toxic state [MAS (III)]; thus, the prevalence of *ars*M needs more attention [26,27]. 

### 3.3. Characterization of Bacterial Communities in Henan Section of the Yellow River

**Figure 4:**
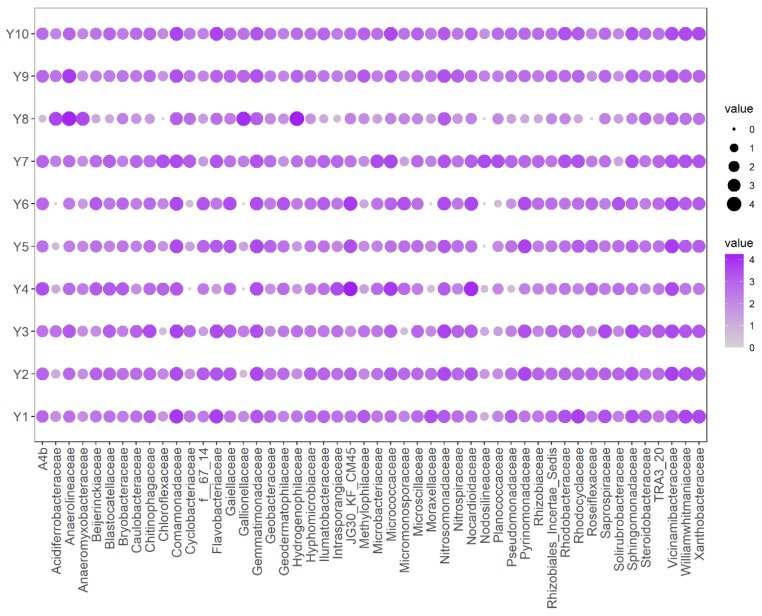
The relative abundance of the top 50 bacteria at the family level. The values in the figure were calculated as follows: log10(bacteria abundance × 10^5^).

The bacterial community at the family level is demonstrated in Figure 4. The average percentage of the top 50 bacteria ranged from 0.37% to 3.7%. The overall detection rate of these bacteria is 98%, which indicates their prevalence. The average variation coefficient (CV%) of these bacteria is 117.34%, which shows that there is variation in the abundance of bacteria among sample sites. 

*Comamonadaceae* is the most abundant bacteria, with an average relative abundance of 3.7%. Moreover, the relative abundance of this bacteria is in the top 10 in all samples in the Yellow River, indicating the stable functions of these genera in the Yellow River. The metabolic capabilities of *Comamonadaceae* could help them span a wide variety of cycles, such as organotrophs, denitrifiers, hydrogen oxidizers, photoheterotrophs, etc. [28]. Most genera of the family have aerobic heterotrophic bacteria metabolism but are capable of switching to other metabolic forms with the change in substrate or electron acceptor availability [29]. Bacteria can overcome detrimental environmental change through changing their metabolic ways or genes and exploiting previously unavailable resources [30]. Therefore, *Comamonadaceae* is the dominant family in the Yellow River.

The average relative abundance of *Vicinamibacteraceae* is the second highest in the Yellow River, with a value of 3.3%. Some members of the *Vicinamibacteraceae* family can degrade complex organic compounds and may also have chitin-degrading genes [31]. Moreover, other families such as *Nocardioidaceae* and *Flavobacteriaceae* could degrade toxic pollutants [32]. Although the pollution level of HMs in the Yellow River is low, there is organic pollution in the river. For example, Chen et al. assessed the human health risks of some POPs, such as organochlorine pesticides (OCPs), polychlorinated biphenyls (PCBs), and polybrominated diphenyl ether (PBDEs), and found that PCBs and OCPs in the trunk water of the Yellow River and in soil and maize irrigated with river water pose potential carcinogenic and non-carcinogenic risks [33]. Song et al. determined the content of endocrine-disrupting chemicals (ECDs) in the Yellow River, and found that ECDs existed in the river (Zhengzhou section) and could pose reproductive risks and developmental impairment to fish in the Yiluohe and Qinhe cross-sections [34]. Feng et al. determined the content of antibiotics in the Yellow River, and detected high concentrations of norfloxacin, carbamazepine, and 5,5-diphenylhydantoin [35]. Pei et al. measured the content of 14 polybrominated diphenyl ethers (PBDE) and found that PBDE contamination in the water of the Yellow River was relatively high [36]. In conclusion, the occurrence of organic pollutants may contribute to the composition of the bacterial community in the Yellow River. 

### 3.4. Relationship between HM Fractions and MRGs

The B_1_, B_2_, and B_3_ fractions of HMs are collectively referred to as available HMs, which could be utilized by bacteria. The Mantel test is an analysis method based on a distance matrix, which can comprehensively reflect the impact of a group of dependent variables on the target indicators. In recent years, the Mantel test has been widely used to analyze the influencing factors of target objects in the study of the environment and ecology [37,38,39]. In this study, the Mantel test was used to comprehensively analyze the correlation between available fractions and MRGs, and the total content of HMs and MRGs in the Yellow River. The results showed that both the available fractions (r = 0.08327, *p* = 0.2895, permutations = 9999) and the total content (r = 001713, *p* = 0.4206, permutations = 9999) of HMs are not related to MRGs. We further analyzed the relationship between the indexes of MRGs and the HMs by adopting Spearman’s analysis (Table S1), and found that very limited MRG indexes had a significantly positive correlation with bioavailable fractions (six MRGs) and total content (six MRGs) of the HMs. These results indicate that both the bioavailable fractions and the total content of HMs could have little influence on MRGs in the Yellow River.

HMs could put selective pressure on MRGs in HM-contaminated environments. Our previous study indicated that the B1 fractions could influence MRG content in the presence of the fraction occupied by a certain proportion of HMs [16]. However, the B4 faction, which could not be absorbed by bacteria, is the main contributor (71.04%) of the total content of HMs in the Yellow River. Although HMs are cytotoxic and exert mutagenic and carcinogenic properties beyond permissible levels, many of them (such as Cu, Ni, and As) are essential for bacterial growth at low content. Thus, HMs play both stimulatory and inhibitory functions in bacteria growth. In conclusion, low-content HMs may have little influence on MRGs [40]. 

### 3.5. Co-Occurrence Pattern between MRGs and Bacterial Community Composition in the Yellow River 

The co-occurrence patterns among the MRGs and bacterial community structure were determined by co-occurrence network analysis based on a robust (r > 0.6) and significant (*p* < 0.05) Spearman correlation result, and the nodes in the identical module were more related to each other than the nodes from other modules. The co-occurrence pattern of MRGs is shown in Figure S1. The MRGs are divided into eight modules, and the MRG number of each module ranges from three to nine. The connections between MRGs indicate that these genes may be located in the same genetic elements or carried by specific bacteria. Furthermore, many of the MRGs that conferred resistance to the same HMs usually belong to the same module, meaning that they share a similar fate in the Yellow River.

**Figure 5:**
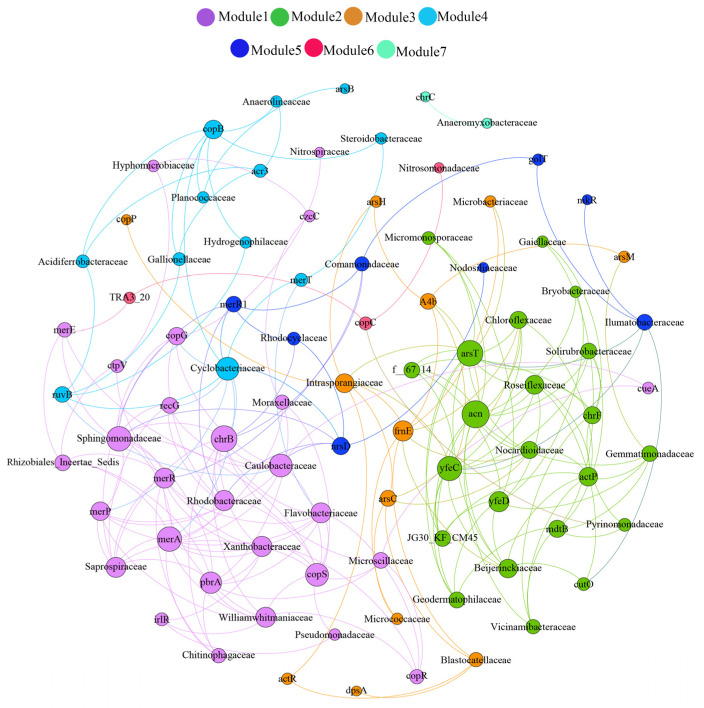
Network analysis revealing co-occurrence patterns among MRGs and bacteria (family level). Each connection represents a strong (r > 0.6) and significant (*p* < 0.05) relationship. The node size is proportional to the connection number—the larger the connection number, the larger the node size.

The co-occurrence network among MRGs and bacteria can be classified into seven modules based on topology, and the node number of each module ranges from 3 to 28 (Figure 5). Module 1 is the largest module, which contains 13 MRGs and 14 bacteria. Previous studies have suggested that co-occurrence patterns between functional genes (such as MRGs and ARGs) and bacterial communities could indicate possible host information of genes [41,42]. *Pseudomonadaceae*, *Planctomycetaceae,* or *Rhodobiaceae* are the potential hosts of merP, and *Helicobacteraceae* is the potential host of *acr*A-01 and *aad*A2-01. Some MRGs of module 1, such as *mer*P, *cop*R, *cop*S, and *mer*A, are prevalent in the Yellow River, and the various potential hosts of these genes may be one of the important reasons for this prevalence. Similar to MRGs, 7 out of 14 bacteria of module 1 are prevalent (top 20) in the Yellow River, which means the prevalent bacteria may carry various MRGs. Previous studies indicated that MRGs can transform and modify the physicochemical characteristics of metals, leading to an alteration in their speciation, mobility, and toxicity [43]. For example, *mer*A and *mer*P can help reduce Hg^2+^ to Hg^0^, which has stronger volatility and toxicity [44]. Thus, the occurrence of MRGs in prevalent bacteria may increase the human health risk.

Module 2 is the second-largest module, which contains 13 bacteria and 9 MRGs. The bacteria (such as *Vicinamibacteraceae*, *JG30_KF_CM45* and *Gemmatimonadaceae*) of module 2 are prevalent, and the MRG content of the module is less than that of Module 1. Moreover, the number of prevalent bacteria and MRGs of module 3 (the third-largest module, containing four bacteria and eight MRGs) and module 4 (the third biggest module, six bacteria and six MRGs) is less than that of Module 1. Thus, these modules contribute less to the HM transformation than Module 1.

## 4. Conclusions

1. Copper resistance genes are the most common genes in the Yellow River sediments of the Henan section. In particular, more attention should be paid to the prevalence of the *ars*M gene.
2. The presence of organic pollutants such as antibiotics, ECDs, and OCPs was closely related to the prevalence of *Vicinamibacteraceae*, *Nocardioidaceae,* and *Flavobacteriacea.*
3. Both the bioavailable fractions and the total content of HMs have little influence on MRGs in the Henan section of the Yellow River.
4. Some dominant bacteria are potential hosts of some prevalent MRGs, which may facilitate the propagation of MRGs and have an adverse impact on human health.

## 5. The Limitation of This Study

In this study, we determined the pollution level of MRGs in the Henan section of the Yellow River and evaluated the role of HMs in MRG propagation. Furthermore, network analysis was conducted to determine the co-occurrence pattern between MRGs and bacteria. At a modest level, both the bioavailable fractions and the total content of HMs have little influence on MRGs. However, knowledge of the relationship between heavy metal resistance genes and heavy metals under complex environmental conditions is still limited. Further research should address the contribution of both bioavailable fractions and the total content of HMs to MRGs in highly HM-polluted sediments. 

## Data Availability

The data presented in this study are available on request from the corresponding author.

## Supplementary Materials

The following supporting information can be downloaded at: https://www.mdpi.com/article/10.3390/ijerph191710724/s1, Figure S1: Co-occurrence pattern among MRGs; Table S1: Correlation analysis results between HMs and MRGs.
