# Supplementary material for: Distribution Pattern and Influencing Factors of Heavy Metal Resistance Genes in the Yellow River Sediments of Henan Section
Source: Int J Environ Res Public Health. 2022 Aug 28;19(17):10724. doi: 10.3390/ijerph191710724 (PMC9517883; doi:10.3390/ijerph191710724)
Supplement: Supplementary file 1 [file ijerph-19-10724-s001.zip › ijerph-1825122-supplementary.pdf]

Supporting information for "Distribution pattern and influencing factors of heavy metal resistance genes in the Yellow River sediments of Henan Section"

Kai Zhang[1,2], Kuangjia Li[3], Minghui Tong[1,2], Yangchun Xia[1,2], Yongxin Cui[1,2], Ziyi Liu[1,2], Qi Chen[1,2], Qidi Li[1,2], Feiyue Hu[1,2], Penglin Fan[1,2], Fengxia Yang[4]

1. School of Geographic Sciences, Xinyang Normal University, Xinyang 464000, China

2. Henan Key Laboratory for Synergistic Prevention of Water and Soil Environmental Pollution, Xinyang Normal University, Xinyang 464000, China

3. Development Research Center, Ministry of Water Resources of People's Republic of China, Beijing 100032, China

4. Agro-Environmental Protection Institute, Ministry of Agriculture and Rural Affairs, Tianjin, 300191, China

Text S1 Detailed information for 16S amplicon sequencing

The primers were 338F (5'-ACTCCTACGGGAGGCAGCAG-3') and 806R (5'-GGACTACHVGGGTWTCTAAT-3'). For PCR, 4 μL 5× TransStart FastPfu buffer, 0.8 μL forward primer, 0.8 μL reverse primer, 2 μL 2.5mM dNTPs, 0.4 μL TransStart FastPfu DNA polymerase, 10 ng template DNA were mixed together, then ddH_2O was added to guarantee the reaction system was 20 μL. PCR was performed with a PCR instrument (ABI GeneAmp® 9700, USA) and the following reaction program was set as follows: initial denaturation at 95 °C for 3 min, followed by 27 cycles consisting 95 ℃ for 10 s, annealing at 55 °C for 30 s, extension at 72 ℃ for 30 s, and a final extension at 72 °C for 10 min. Each reaction was run in triplicate for each sample. PCR products were examined by 2% agarose gel electrophoresis and then purified by DNA Gel Extraction Kit (Axygen Biosciences, Union City, CA, USA). Library was constructed by NEXTflexTM Rapid DNA-Seq Kit (Bioo Scientific, USA), then sequencing was performed at Majorbio Bio-Pharm Technology Co., Ltd. (Shanghai, China) , and an Miseq PE300 platform (Illumina, USA) was adopted for sequence analysis. Low-quality bases (length <50 bp, quality value < 20 or presence of N bases) were discarded using Sickle (https://github.com/najoshi/sickle).

Table S1 Correlation analysis results between HMs and MRGs

	Bio_Zn	Bio_Pb	Bio_Cd	Bio_Ni	Bio_Mn	Bio_Cu	Total_Zn	Total_Pb	Total_Cd	Total_Ni	Total_Mn	Total_Cu
acr3	.779**	0.533783	0.34972	0.570595	0.546053	.706*	0	0	0	0	0	0
aioA	-0.0303	0.078788	0.406061	-0.00606	0.006061	0.054545	0	0	0	0	0	0
aioB	-0.36893	-0.3064	0.118808	-0.33141	-0.28764	-0.21886	0	0	0	0	0	0
aioE	-0.00647	-0.21335	-0.278	-0.30386	-0.23921	-0.04526	0	0	0	0	0	0
arrA	0.563636	.721*	.709*	.709*	.685*	.636*	.794**	.721*	0	.770**	0	.733*
arrB	0.38144	0.446091	.731*	0.446091	0.510741	.666*	0	0	0	0	0	0
arsC	-0.41818	-0.47879	-0.50303	-0.49091	-0.6	-.721*	0	0	0	-.648*	0	0
arsH	-0.10303	-0.27273	-0.55152	-0.34545	-0.38182	-0.33333	0	0	0	0	0	0
arsM	0.127273	0.272727	0.357576	0.187879	0.139394	0.054545	0	0	0	0	0	0
arsT	-0.46667	-0.4303	-0.26061	-0.49091	-0.52727	-0.49091	0	0	0	0	-.648*	0
pstB	0.284848	0.454545	0.309091	0.490909	0.442424	0.284848	0	0	0	0	0	0
arsB	.705*	0.480245	0.541036	0.462008	0.437692	.644*	0	0	0	0	0	0
frnE	-0.46898	-0.63156	-0.47523	-.657*	-.750*	-.694*	0	0	0	-.782**	-.725*	-.775**
irlR	0.054545	-0.01818	-0.16364	-0.09091	-0.01818	0.115152	0	0	0	0	0	0
czcA	0.031265	-0.13757	-0.27513	-0.17509	-0.08129	0.093796	0	0	0	0	0	0
czcC	-0.09091	-0.12727	-0.15152	-0.2	-0.12727	0.066667	0	0	0	0	0	0
actR	-0.22532	-0.38919	-0.5394	-0.34139	-0.36871	-0.35505	0	0	0	0	0	0
chrA	0.139394	0.393939	0.381818	0.418182	0.442424	0.321212	0	0	0	0	0	0
chrC	.879**	.952**	0.612121	.927**	.915**	.818**	.867**	.830**	-0.34545	.782**	.939**	.891**
chrF	-0.43146	-0.11255	0.006253	-0.15007	-0.1063	-0.25012	0	0	0	0	0	0

chrB	-0.40967	-0.51892	-.642*	-0.43016	-0.35505	-0.32091	0	-.676*	0	0	0	0
recG	-.662*	-.785**	-.847**	-.744*	-.683*	-.662*	-.860**	-.860**	0	-.758*	0	-.737*
ruvB	-0.01841	-0.16566	-0.33745	-0.06749	-0.01841	-0.00614	0	0	0	0	0	0
cmtR	0.170697	-0.02731	-0.19118	-0.02731	-0.04097	0.081935	0	0	-.874**	0	0	0
copB	0.624242	0.393939	0.187879	0.406061	0.454545	0.612121	0	0	0	0	0	0
copC	-.685*	-0.57576	-0.47879	-0.55152	-0.52727	-.697*	0	0	.636*	0	0	0
copF	0.212121	0.272727	0.2	0.236364	0.284848	0.345455	0	0	0	0	0	0
copG	-0.54953	-.718*	-.782**	-.679*	-0.61418	-0.54953	-.795**	-.834**	0	-.692*	0	-.666*
copP	0.056277	-0.21886	-0.38144	-0.23136	-0.25638	-0.0938	0	0	0	0	0	0
copR	-0.2	-0.17576	-0.45455	-0.15152	-0.09091	-0.17576	0	0	0	0	0	0
copS	-0.32121	-0.45455	-.661*	-0.47879	-0.40606	-0.32121	0	0	0	0	0	0
cop_unnamed	0.345455	0.587879	.685*	0.527273	0.50303	0.393939	.733*	.733*	0	.685*	0	.648*
corR	.806**	0.58037	0.443813	0.58037	0.518919	.683*	0	0	0	0	0	0
ctpG	0.393939	0.466667	0.381818	0.393939	0.357576	0.333333	0	.685*	0	0	0	0
ctpV	-0.08511	-0.13374	0.139818	-0.18237	-0.07903	0.206688	0	0	0	0	0	0
cutO	-.661*	-0.52727	-0.34545	-0.56364	-0.50303	-0.50303	0	0	0	0	0	0
golT	-0.60125	-0.5366	-0.18749	-0.48488	-0.42023	-0.36851	-0.61418	-.653*	0	0	0	0
copA	-0.04242	-0.2	0.018182	-0.24848	-0.21212	-0.0303	0	0	0	0	0	0
cueA	-0.32518	-0.46016	-.681*	-0.42334	-0.46016	-0.53378	0	0	0	0	0	0
actP	-0.35758	-0.27273	-0.32121	-0.29697	-0.39394	-0.58788	0	0	0	0	0	0
acn	-0.4303	-0.35758	-0.27273	-0.4303	-0.49091	-0.53939	0	0	0	0	0	0
dpsA	-0.49091	-0.51515	-.782**	-0.46667	-0.50303	-.697*	0	0	0	0	0	0

yfeC	-0.54711	-0.4924	-0.31003	-0.54104	-0.57751	-0.57143	0	0	0	0	-.687*	0
yfeD	-0.20061	-0.04863	0.200609	-0.08511	-0.13374	-0.17629	0	0	0	0	0	0
pbrA	-0.32091	-0.47112	-0.60768	-0.51209	-0.43698	-0.25946	0	0	0	0	0	0
merE	-.700*	-.744*	-0.53151	-.682*	-0.6128	-0.56277	-.794**	-.838**	0	0	0	-.713*
merP	-0.35758	-0.50303	-.636*	-0.46667	-0.3697	-0.29697	0	-.661*	0	0	0	0
merR	-0.28485	-0.3697	-0.29697	-0.35758	-0.24848	-0.04242	0	0	0	0	0	0
merR1	-0.46977	-0.52942	-0.6189	-0.45486	-0.36538	-0.35046	-.664*	-.664*	0	0	0	0
merT	0.066667	-0.00606	-0.30909	0.066667	0.163636	0.175758	0	0	0	0	0	0
merT_P	0.418182	0.454545	0.2	0.406061	0.454545	0.527273	0	0	0	0	0	0
merA	-0.38182	-0.61212	-.661*	-0.6	-0.50303	-0.29697	-.661*	-.745*	0	0	0	0
nikR	-0.27312	-0.20484	0.163869	-0.20484	-0.12973	-0.00683	0	0	0	0	0	0
nrsD	-0.18749	-0.38144	-0.47195	-0.30386	-0.25214	-0.17456	0	-.640*	0	0	0	0
mdtB	-0.48488	-0.18749	0.058186	-0.21335	-0.21335	-0.36851	0	0	.666*	0	0	0

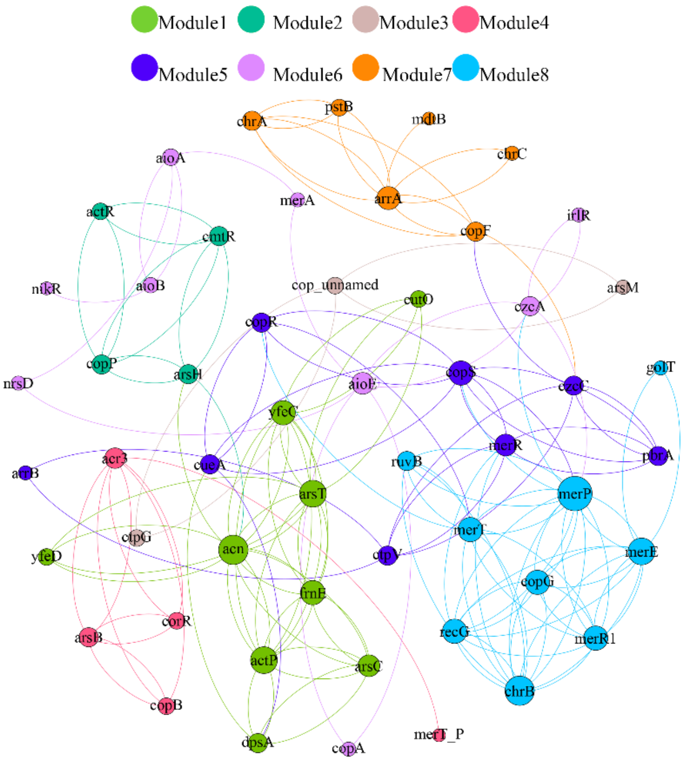

Figure S1 Co-occurrence pattern among MRGs
